# Functional Annotation of FD Cluster Phage Bouclier that Infects *Arthrobacter globiformis*

**DOI:** 10.17912/micropub.biology.002177

**Published:** 2026-06-04

**Authors:** Matias Espinosa, Hannah Atchley, Kaden K Bambach, Benjamin Biggs, Onyx Barnes, Ethan W Brown, Kelis Brown, Brianna Bruni, Phuong Dang, Ashe Dickerson, Kaylee Dunnigan, Riya George, Victoria Hiscock, Mehalia Hoyte, Ella King, Christopher Kosenko, Bianca Kulcsar, Duong Khanh Vinh Nguyen, Khai Thanh Nguyen, Olivia Payne, Zayda Perez Pedraza, Richard Quinones, Mina Takla, Richard S Pollenz

**Affiliations:** 1 Molecular Biosciences, University of South Florida, Tampa, FL, US

## Abstract

Phage Bouclier has a 19,912bp genome with 29 protein coding genes and infects *Arthrobacter globiformis* B-2979. Transmission electron microscopy visualizes Bouclier as a podovirus. This is confirmed by identification of genes encoding structural proteins found in other podoviriruses. The portal, upper collar, tail knob, and major capsid proteins can be modeled using AlphaFold3 into macromolecular structures. These structural components have high confidence matches in ChimeraX to Bacillus phage Φ29 that has been resolved by cryoEM. Bouclier contains a defined lysis cassette including two genes encoding Lysin A proteins and three putative transmembrane proteins that may be involved in host lysis.

**Figure 1. Modeling of Bouclier Structural proteins using AlphaFold3 and ChimeraX and summary of data:** A) The Bouclier genomic structure and location of the structural and predicted lysis genes. B) AlphaFold3 models of Bouclier (teal) and Φphi29 (red) structural proteins and the alignment of the structures using Matchmaker in ChimeraX. Scaffolding modeled as a monomer (1x); major capsid as pentamer (5x); tail knob as a hexamer (6x); and portal and lower collar modeled with 12x subunits. Matchmaker alignment statistics are provided in the text. C) AlphaFold3 models of trimers of pre-neck appendage proteins from Bouclier, Φ29 and N4. Table 1: Summary of Bouclier and Φphi29 structural protein data elements.

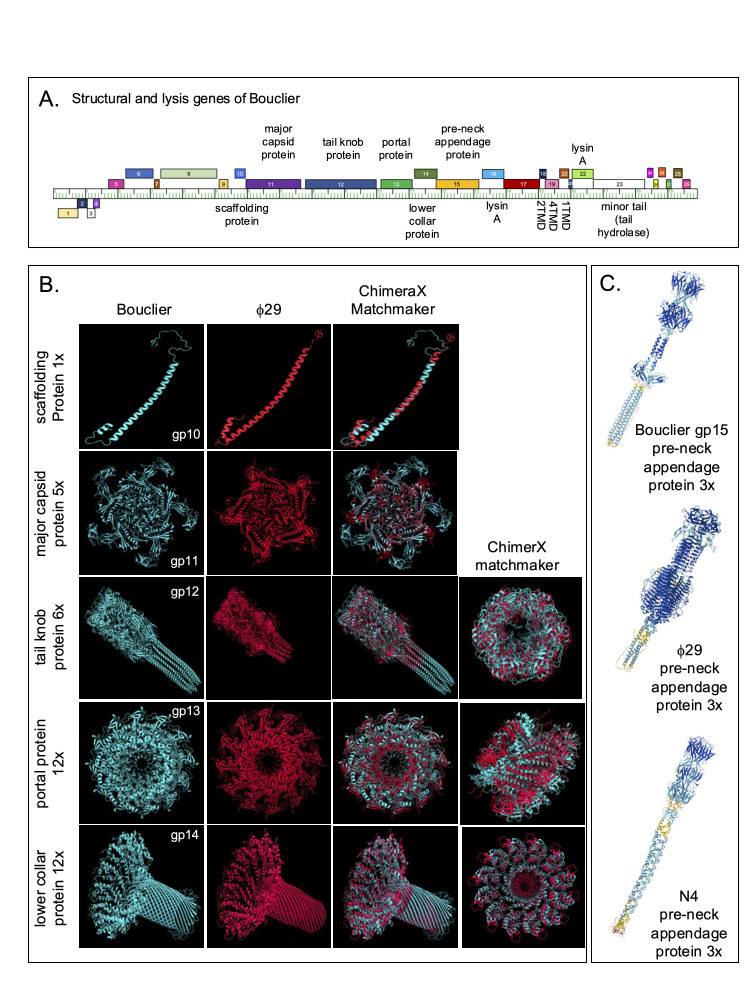



**Table:** 

Protein Function	Bouclier gene number	phi29 gene number	Bouclier protein size (amino acid)	phi29 protein size (amino acid)	# of subunits in assemby	Bouclier and phi29 % amino acid identity	% of atoms used in match	Matchmaker RMSD values (angstroms)
scaffolding protein	10	7	104	98	1	21.1	55	1.183
major capsid protien	11	8	577	488	5	38.7	65	1.035
tail knob protein	12	9	732	598	6	31.1	33	1.177
portal protein	13	10	319	309	12	31.1	65	0.864
lower collar protein	14	11	229	293	12	40.5	64	0.882
pre-neck appendage protein	15	12	442	854	3	19.8	9.8	0.977
tail hydrolase (minor tail)	23	13	540	364	1	17.1	ND	ND

## Description

The identification of novel phages has the potential to uncover new genes that are involved in immunity, defense and host lysis that can help in the characterization of phage candidates for use in agriculture, health and food safety (Shield et al., 2021; Wang and Zhao, 2022; Strathdee et al., 2023).

Bouclier was isolated from ~12.5g moist soil in Tampa, FL (28.06204 N, 82.41601 W). Soil was mixed with equal parts peptone-yeast calcium medium (PYCa), shaken at 250rpm for 2 hr. at 25 ^o^ C and filter sterilized (0.2µm PES). 500ul of the sterile soil filtrate was mixed with 250ul of saturated *Arthrobacter globiformis* NRRL B-2979 and plaque assays performed by mixing the sample with 5ml of PYCa top agar and plating on PYCa agar plates. Plates were incubated at 30 ^o^ C for 24-36 hrs. and cloudy plaques of ~2mm were observed. A single plaque separated 2cm from others was picked with a sterile 1000ul pipette tip, resuspended in 100ul of Phage Buffer (PB; 10mM Tris pH 7.5; 1mM CaCl2; 10mM MgSO4; 68mM NaCl), vortexed and then serial diluted in PB. After a 10-minute incubation at 22 ^o^ C, 10ul aliquots were used to complete plaque assays as described above. A plaque of similar shape and size to the original was picked from the plate, serial diluted and plated as above. After confirming that all resulting plaques on the plates were of uniform plaque morphology, plates with near full lysis were flooded with 5ml of PB, incubated for 6 hrs. and the PB filter sterilized (0.2µm PES) to generate a high titer filtrate. The resulting filtrate was serial diluted to determine the titer and genomic DNA isolated using the Wizard DNA clean-up kit (A7280; Promega). DNA samples were processed using the NEB Ultra II Library Kit and the sequence determined by the Pittsburgh Bacteriophage Institute using an Illumina NextSeq 1000 instrument. 59M bases were evaluated from 606,352 spots resulting in 2,963 shotgun coverage. Raw reads were trimmed with cutadapt 4.7 (using the option: –nextseq-trim 30) and filtered with skewer 0.2.2 (using the options: -q 20 -Q 30 -n -l 50) prior to assembly. The resulting sequences were assembled using Consed (v29.0) with Unicycler (v5.0) and contigs checked for completeness, accuracy, and genome termini (Gordon et al., 1998; Russell, 2018). Default parameters were used for all software. The Bouclier genome ends have a covalent terminal protein and the genome was bioinformatically linearized such that base 1 is assigned in accord with other *Arthrobacter* phages (Russell, 2018). Bouclier was auto-annotated using DNA master (v5.23.6) (Pope and Jacobs-Sera, 2018) and the genes then manually validated for starts and functional calls. GeneMark (v2.5) (Besemer and Borodovsky, 2005) and Glimmer (v3.02) (Delcher et al., 2007) were utilized to assess start sites and coding potential. Starterator (v601) (github.com/SEA-PHAGES/starterator) to summarize the starts across each family of phage genes. Evidence to support a gene product function was collected using HHpred (databases: PDB_mmCIF70_30_Mar; Pfam-A_v37; NCBI_Conserved_Domains(CD)_v3.19) (Söding, 2005; Marchler-Bauer, 2015), and NCBI BLAST (BLAST+2.13) (Altschul et al., 1990). Putative transmembrane domains (TMD) were identified using Deep TMHMM (v1.0.24) (Hallgren et al., m2022) and TOPCONS (v2.0) (Tsirigos et al, 2015). Information on Bouclier regarding isolation, characterization, and gene content is archived in Phamerator (Actino_draft database v578; Cresawn et al., 2011), and the Actinobacteriophage Database at PhagesDB.org (https://phagesdb.org/phages/Bouclier).

Bouclier is grouped to Cluster FD in the Actinobacteriophage database (PhagesDB: https://phagesdb.org; Pope et al., 2017; Russell and Hatfull, 2017). Bouclier is a podovirus as determined by negative stain TEM (https://phagesdb.org/phages/Bouclier/) and has an icosahedral capsid with a diameter of ~40nm and no obvious tail. The proteins encoded by Bouclier were analyzed using HHpred, and only PDB hits with probabilities >90% and E-values <10-3 were considered for functional annotation. 19 of the 29 gene products could be sorted into five general categories: host lysis (gp16, gp18, gp19, gp20, gp22); enzymes and DNA binding (gp1, gp5, gp6, gp8, gp9, gp17); structural (gp10-gp15 and gp23; Figure 1A ) and an unclassified lipoprotein (gp25). It was hypothesized that AlphaFold3 (Abramson et al., 2024) and ChimeraX/Matchmaker (Meng et al., 2006; Meng et al., 2023) could be utilized to model the Bouclier structural proteins and compare the macromolecular assemblies to the resolved cryoEM structural proteins of Bacillus phage Φ29 (Xu et al., 2018) to strengthen the functional annotation. Φ29 is a podovirus that infects the Gram-positive bacteria *Bacillus subtilis* yet it shares a similar set of structural proteins with Bouclier that include: scaffolding protein, major capsid protein, portal protein, lower collar protein, tail knob protein, pre-neck appendage protein and a putative tail hydrolase that are in the same general genomic organization (Xiang et al., 2008; Xu et al., 2018). Φ29 also encodes an SSB protein, DNA terminal protein, DNA polymerase and a terminase like Bouclier. To validate the predicted structural proteins of Bouclier, they were modeled in AlphaFold3 and the monomers assembled into macromolecular structures based on the number of subunits defined by the Φ29 cryoEM data (Xu et al., 2018). The macromolecular assemblies were exported from AlpahFold3 and evaluated for structural similarity by ChimeraX using Matchmaker with the default parameters. Matchmaker output provides root-mean-square deviation (RMSD) data based on the proximity of the atoms that have been aligned between two structures (Meng et al., 2006). Highly similar structures have RMSD values of <2.0 angstroms and with a majority (>50%) of the atoms in each structure being aligned to a similar space. The Chimera X results are presented in Figure 1B, 1C and the data is summarized in Table 1. Bouclier is colored teal and Φ29 in red. Both phages encode a scaffolding protein that models as a monomer. There is good alignment with 55% of the atoms used and an RMSD of 1.183 angstroms. Φ29 major capsid protein forms pentameric and hexameric capsomers to form the prolate capsid structure (Xu et al., 2018). Bouclier gp11 and Φ29 major capsid proteins were modeled as pentamers and hexamers and the pentameric capsomers are shown in Figure 1B . Matchmaker shows alignment with 65% of atoms used and the RMSD of 1.035 angstroms. gp12 of Bouclier is the predicted tail knob and although only 33% of the atoms were used when compared to the Φ29 tail knob, the RMSD is 1.177 angstroms supporting a similar core structure. The portal and lower collar proteins assemble as dodecamers that connect to form the base of the phage structure. When evaluated in Matchmaker, gp13 of Bouclier and Φ29 portal protein have an RMSD of 0.864 angstroms with 65% of the atoms aligned. The lower collar proteins used 64% of the atoms with an RMSD of 0.882 angstroms. The pre-neck appendage protein forms twelve trimers that attach to the lower collar/portal interface near the base of the capsid and provide the host recognition sites (Xu et al., 2018). The trimers for each are shown in Figure 1C along with the pre-neck appendage protein from podoviridea phage N4 that infects *E. coli* (Zheng et al., 2024). Although these three proteins share <20% amino acid identity, they each model as a trimer with an elongated structure that has a globular head region for attachment to the main phage structure and extended alpha helical tails that extend out for binding to the host bacteria. ChimeraX failed to align >10% of the atoms. Finally, both Bouclier and Φ29 encode an additional tail structural protein. Φ29 encodes a tail hydrolase (gp13; Xiang et al., 2008) and Bouclier gp23 encodes a 540aa protein that is not found in any other actinobacteriophages but has predicted hydrolase activity based on numerous HHpred hits to esterases and hydrolases from bacterial species. AlphaFold3 also shows that both proteins bind Zn ^2+^ ions (ipTM scores >0.85) typical for many enzymes. The Φ29 tail hydrolase protein is predicted to associate with distal end of the tail knob in Φ29 (Xiang et al., 2008) but was not resolved in the cryoEM work by Xu et al., 2018. The strength of the Bouclier modelling data has refined the functional annotation of the FD phages. Collectively, these findings suggest that small podoviruses infecting diverse hosts may use a conserved structural protein set, supporting evolution from a common ancestor.

Bouclier and Φ29 differ in the encoded proteins predicted to be involved in host lysis following infection ( Figure 1A ). Φ29 encodes a Lysin A and a type I holin similar to the R and S proteins encoded by phage lambda (Young 2014; Cahill and Young 2019). In contrast, Bouclier gp16 encodes a Lysin A enzyme with M23 peptidase catalytic activity while gp22 encodes a Lysin A with N-acetylmuramoyl-L-alanine amidase activity. Recent studies have shown that actinobacteriohages encode several distinct TMD proteins that have different functions during the lysis event (Pollenz and Bland 2022; Pollenz et al., 2026). Bouclier encodes a 2TMD protein (gp18) and a 1TMD protein (gp20) that are similar in topology to the LysF1a and LysF1b lysis regulators classified in Mycobacteriophage Girr where the LysF1b provides the primary lysis function (Pollenz et al., 2026). Bouclier also encodes a novel four-TMD protein (gp19) that may also participate in the lysis pathway due to its location adjacent to genes *18* and *20* .
